# Low bone turnover is associated with plain X-ray vascular calcification in predialysis patients

**DOI:** 10.1371/journal.pone.0258284

**Published:** 2021-10-13

**Authors:** Ricardo Neto, Luciano Pereira, Juliana Magalhães, Janete Quelhas-Santos, João Frazão

**Affiliations:** 1 Institute for Innovation and Health Research (I3S), Institute of Biomedical Engineering (INEB), Nephrology and Infectious Diseases Research Group, University of Porto, Porto, Portugal; 2 Department of Nephrology, Centro Hospitalar Universitário São João, Porto, Portugal; 3 Faculty of Medicine, University of Porto, Porto, Portugal; University of Liège, BELGIUM

## Abstract

**Background:**

Vascular calcification (VC) is a common finding in chronic kidney disease (CKD) patients and predicts subsequent cardiovascular morbidity and mortality in this population. Vascular calcification is linked to disordered mineral metabolism and has been associated with bone histomorphometry changes in CKD. However, data on predialysis patients is scarce.

**Methods:**

A cross-sectional study was conducted on a cohort of 56 CKD patients not yet on dialysis, who underwent a transiliac bone biopsy for histomorphometric evaluation after double tetracycline labeling. Patients had no previous exposure to calcium salts, vitamin D agents, steroids or bisphosphonates. Vascular calcification was assessed at the time of biopsy, using Kauppila (plain X-ray of the lateral lumbar spine) and Adragão (plain X-ray of the pelvis and hands) scores.

**Results:**

Vascular calcification was seen in two-thirds of the cohort. Subjects with VC were more likely to be male and have diabetes, and had significantly higher sclerostin and osteoprotegerin circulating levels than those without VC. The histomorphometric analysis showed that bone formation rate was significantly lower in VC compared to non-VC patients. In the multivariable logistic regression analysis, bone formation rate was independently associated with the presence of VC.

**Conclusions:**

Vascular calcification is highly prevalent in predialysis patients, especially in those with diabetes. The independent association between bone formation rate and VC provides evidence of an important interaction between bone and vessel in CKD. Our results suggest that low bone turnover is a non-traditional risk factor for cardiovascular disease in predialysis patients.

## Introduction

Chronic kidney disease (CKD) is associated with a high cardiovascular risk, but traditional factors, such as diabetes, hypertension, smoking and dyslipidemia, do not fully explain this association [1]. Vascular calcification (VC) is a common finding in CKD patients and its prevalence increases with disease progression [2]. The presence of VC is a well-known predictor of cardiovascular morbidity and mortality in the general population and in patients with CKD [3]. Several types of VC can occur simultaneously in CKD patients: intimal calcification of atherosclerotic plaques, arterial media calcification (arteriosclerosis or Monckeberg’s disease) and cardiac valvular calcifications. Medial calcification is relatively specific to CKD because it associates with abnormal mineral and bone metabolism [4]. Vascular calcification is an active cell-mediated event that resembles normal osteogenesis in several aspects [5–7]. Current guidelines suggest screening for VC occurrence in order to inform therapeutic management [8].

The existence of a link between bone and vascular tissue metabolism is supported by bone biopsy studies. In dialysis patients, low bone activity [9, 10] and low bone volume [11, 12] have been associated with cardiovascular calcifications. However, data on CKD stages 3 and 4 is very limited [13].

The purpose of this cross-sectional study was to evaluate the relationship between bone histomorphometry and VC assessed by plain X-ray in a cohort of patients not yet on dialysis.

## Materials and methods

### Patients and study design

A cross-sectional observational study was conducted on a cohort of 56 patients from our predialysis clinic. Subjects were recruited between February 2014 and September 2019. Inclusion criteria were capacity to give written informed consent, 18 years of age or older and glomerular filtration rate (GFR) between 15 and 60 mL/min/1.73m^2^. Patients were excluded in cases of chronic inflammatory or autoimmune disease; active malignancy; parathyroidectomy; oral anticoagulants use, and previous exposure to drugs known to affect mineral and bone metabolism, such as calcium or aluminum salts, native vitamin D or its active analogues, steroids or bisphosphonates. Clinical characteristics were collected for all patients, including age, sex, CKD etiology, presence or absence of diabetes mellitus, body mass index and blood pressure. The study protocol was approved by the Ethics Committee of Centro Hospitalar Universitário São João (CES 299–13).

### Biochemistry tests

Blood samples were collected in a fasting state at the time of bone biopsy. Assessment of serum calcium, albumin, creatinine, phosphorus and alkaline phosphatase, together with urine calcium, creatinine and phosphorus, were performed using an Olympus AU5400 (Olympus America Inc., Center Valley, PA, USA). Serum intact PTH (iPTH) and 25-hydroxyvitamin D [25(OH)D] levels were measured through an electrochemiluminescence immunoassay with a Cobas E411 analyzer (Roche Diagnostics GmbH, Mannheim, Germany). Serum bicarbonate was determined with a Siemens RapidLab 1265 gas analyzer (Siemens Healthcare Diagnostics, Tarrytown, NY, USA). Bioactive sclerostin and free soluble receptor activator of nuclear factor-κB ligand (RANKL) were measured in plasma samples by an enzyme immunoassay (Biomedica Medizinprodukte GmbH, Wien, Austria), according to the manufacturer’s instructions. The detection range of the assays are 1.9–320 pmol/L and 0.2–40 pg/mL, respectively. Osteoprotegerin (OPG), Dickkopf-1 (DKK1) and intact fibroblast growth factor 23 (FGF23) were measured in plasma samples by a multiplex assay (Magnetic Luminex Assay, R&D Systems Inc., Minneapolis, MN, USA), according to the manufacturer’s protocol. Detection range of the assay for each of the analytes is 75–18220 pg/mL, 202–49060 pg/mL and 11.8–2870 pg/ml, respectively. GFR was estimated using Chronic Kidney Disease Epidemiology Collaboration (CKD-EPI) equation [14]. CKD stages were defined according to Kidney Disease: Improving Global Outcomes (KDIGO) classification guideline [15].

### Bone histomorphometry

Anterior iliac crest bone biopsies were performed after double tetracycline labeling (doxycycline 200 mg twice daily for 3 days, repeated after an interval of 12 days), using a modified Bordier trephine. Biopsy samples were about 5 mm in diameter and 10 mm in length. Bone was dehydrated in alcohol, cleared in xylene and subsequently embedded in methyl methacrylate. Undecalcified sections were cut at a thickness of 5 μm and stained with modified Masson-Goldner trichrome for histomorphometric measurement of static parameters. Unstained 10 μm sections were prepared for histodynamic evaluation under fluorescence light. OsteoMeasure software (OsteoMetrics, Decatur, GA, USA) was used for histomorphometric analysis. About 30 fields per section were consecutively counted at 20x magnification. Bone histomorphometry units were those suggested by the American Society for Bone and Mineral Research [16]. Reference ranges for static and dynamic parameters were obtained from published literature [17–20]. Normal bone turnover was defined as bone formation rate/bone surface (BFR/BS) of 10.95–37.50 μm^3^/μm^2^/yr. Mineralization was considered normal if mineralization lag time (Mlt) was less than 50 days and osteoid thickness (O.th) was less than 20 μm. Biopsies were evaluated by two different observers, with no knowledge of clinical, biochemical or radiographic findings.

### Radiological assessment

Plain radiographs of the lateral lumbar spine, pelvis and hands were taken at the time of bone biopsy. Vascular calcifications were assessed as proposed by Kauppila *et al*. and Adragão *et al* [21, 22]. Kauppila score estimates lumbar aortic wall calcification and varies from 0 to 24. Adragão score evaluates iliac, femoral, radial and digital arteries calcification, ranging from 0 to 8. Both indices correlate well with coronary artery calcification (CAC) estimation by computed tomography [12, 23]. Kauppila and Adragão scores have been shown to associate with cardiovascular mortality in CKD patients [24–26]. The X-rays were read by two independent observers, blinded to clinical, biochemical and histological information. Inter-rater agreement was evaluated through Coen’s weighted Kappa [27]. Mean scores attributed by the two observers were used for analysis. Vascular calcification was defined as a Kauppila or Adragão score greater than 0.

### Statistical analysis

Since our study was exploratory, all eligible participants in the specified time period were included and, therefore, sample size was not determined in advance. Continuous data were expressed as mean and standard deviation (SD) or median and interquartile range (IQR) depending on variable distribution. Categorical variables were expressed as frequencies and percentages. In order to compare differences between groups, Mann-Whitney test was used for continuous variables and Chi-square test for categorical variables. Spearman’s coefficient was used to assess linear correlations. Distribution of both Kauppila and Adragão scores was markedly skewed and resistant to normalizing transformation. As such, the two variables were grouped dichotomously according to the presence or absence of VC. Multivariable analysis was performed through logistic regression to assess the relationship between bone histomorphometric parameters and VC status. Data analysis was carried out with SPSS version 27 (IBM SPSS Statistics, Chicago, IL, USA). *P* values < 0.05 were considered statistically significant.

## Results

### Clinical, laboratory and histomorphometric characteristics

Demographic, clinical and biochemical characteristics of the 56 patients included in the cohort are presented in Table 1. Subjects were predominantly male (*n* = 44, 78.6%) and all were Caucasian. Mean age was 65.7 ± 9.8 years old. The majority had diabetes (*n* = 35, 62.5%). Most cases of CKD were due to diabetes (*n* = 17, 30.4%), followed by hypertension (*n* = 16, 28.6%) and chronic tubulointerstitial nephritis (*n* = 8, 14.3%). Mean serum creatinine was 2.32 ± 0.43 mg/dL, corresponding to a mean estimated GFR of 27.8 ± 6.8 mL/min/1.73m^2^. Twenty-five patients had stage G3b CKD (44.6%) and 31 had stage G4 CKD (55.4%). Mean serum calcium and phosphorus were 9.3 ± 0.5 and 3.5 ± 0.6 mg/dL, respectively. The vast majority of the patients (n = 51, 91.1%) presented with either vitamin D deficiency (<15 ng/mL) or insufficiency (15–29 ng/mL). Median iPTH and FGF23 levels were 93.3 pg/mL (IQR 50.6–151.4 pg/mL) and 25.5 pg/mL (IQR 16.3–38.4 pg/mL), respectively. Patients with CKD stage 4 had significantly higher brain natriuretic peptide, iPTH and FGF23 levels than those with stage 3. No other bone biomarker differed between CKD stages. Subjects with diabetes had significantly higher phosphate, albumin, C-reactive protein, brain natriuretic peptide and sclerostin levels (61.1 vs 39.3 pmol/L, *P* = 0.035), compared to those without diabetes. No other clinical or biochemical difference was seen between groups (S1 Table).

**Table 1:** Demographic, clinical and laboratory characteristics of the study population according to CKD stage.

		All (*n* = 56)	CKD 3 (*n* = 25, 44.6%)	CKD 4 (*n* = 31, 55.4%)	*P* value
**Clinical**					
	Age (yr)	65.7 (9.8)	63.6 (11.2)	67.6 (8.5)	0.364
	Male (%)	78.6	88.0	71.0	0.191
	Diabetes (%)	62.5	56.0	67.7	0.415
	Body mass index (Kg/m^2^)	28.4 (4.3)	29.3 (4.5)	28.3 (4.4)	0.499
	SBP (mmHg)	136 (11)	134 (10)	137 (12)	0.276
	DBP (mmHg)	78 (8)	79 (8)	81 (8)	0.334
**Biochemistry**					
	Creatinine (mg/dL)	2.32 (0.43)	1.98 (0.22)	2.57 (0.36)	<0.001
	GFR (mL/min/1.73 m^2^)	27.8 (6.8)	34.3 (3.3)	23.0 (4.0)	<0.001
	Haemoglobin (g/dL)	13.0 (1.7)	13.1 (2.1)	13.0 (1.4)	0.856
	Albumin (g/dL)	4.16 (0.35)	4.12 (0.38)	4.19 (0.33)	0.215
	Bicarbonate (mmol/L)	25.2 (3.3)	25.1 (3.5)	25.3 (3.2)	0.721
	C-reactive protein (mg/L)	2.0 (0.8, 6.1)	1.3 (0.7, 6.4)	3.2 (1.5, 5.0)	0.285
	LDL cholesterol (mg/dL)	94 (30)	98 (27)	91 (32)	0.562
	BNP (pg/mL)	43 (21, 106)	29 (17, 54)	54 (43, 144)	0.010
	Calcium (mg/dL)	9.3 (0.5)	9.4 (0.6)	9.3 (0.4)	0.179
	Phosphorus (mg/dL)	3.5 (0.6)	3.4 (0.7)	3.6 (0.5)	0.173
	Magnesium (mEq/L)	1.65 (0.24)	1.63 (0.26)	1.67 (0.23)	0.799
	25(OH)D (ng/mL)	16 (9, 22)	16 (11, 22)	15 (9, 23)	0.611
	ALP (U/L)	81 (63, 102)	68 (55, 99)	81 (64, 110)	0.432
	iPTH (pg/mL)	93.3 (50.6, 151.4)	67.4 (40.3, 101.6)	127.9 (79.4, 211.6)	0.001
	FGF23 (pg/mL)	25.5 (16.3, 38.4)	18.7 (11.3, 27.8)	34.2 (23.3, 48.6)	0.002
	Sclerostin (pmol/L)	57.6 (38.6, 72.8)	60.0 (39.1, 86.5)	47.7 (37.8, 69.2)	0.245
	DKK1 (pg/mL)	800 (276)	851 (356)	753 (170)	0.676
	sRANKL (pg/mL)	2.66 (1.69, 3.58)	2.34 (1.64, 3.07)	2.85 (1.95, 5.15)	0.214
	Osteoprotegerin (pg/mL)	1385 (1120, 1717)	1374 (1053, 1634)	1429 (1132, 1783)	0.416
	Urine calcium (mg/day)^a^	70 (36, 99)	86 (62, 115)	54 (33, 91)	0.077
	Urine phosphate (mg/day)^a^	693 (556, 797)	737 (605, 881)	648 (545, 774)	0.154
	Urinary protein (mg/day)^a^	720 (255, 2287)	420 (210, 1695)	770 (310, 2920)	0.340

CKD, chronic kidney disease; SBP, systolic blood pressure; DBP, diastolic blood pressure; GFR, glomerular filtration rate estimated by CKD-EPI equation; LDL, low-density lipoprotein; BNP, brain natriuretic peptide; 25(OH)D, 25 hydroxyvitamin D; ALP, alkaline phosphatase; iPTH, intact parathyroid hormone; FGF23, fibroblast growth factor-23; DKK1, Dickkopf-1; sRANKL, soluble receptor activator of nuclear factor-κB ligand.

Data are reported as mean (SD) for normally distributed variables, median (interquartile range) for non-normally distributed variables, or percentage for categorical variables. *P* values were calculated using Mann-Whitney test for continuous variables and Chi-square test for categorical variables.

^a^Twenty-four hour urine collections were adjusted for adequacy using urinary creatinine excretion.

The histomorphometric analysis showed that normal bone histology was the most common histological pattern (n = 23, 41%), followed by low bone turnover with normal mineralization (adynamic bone disease) (n = 20, 35.7%) and high bone turnover with normal mineralization (hyperparathyroid bone disease) (n = 12, 21.4%). There was also one patient (1.8%) with high bone turnover with abnormal mineralization (mixed uremic osteodystrophy) and no cases of low bone turnover with abnormal mineralization (osteomalacia) were observed. Patients with CKD stage 4 had a higher relative prevalence of hyperparathyroid bone disease than those with stage 3 (35.5% vs 4.0%, *P* = 0.007). Bone pattern distribution was not significantly different between patients with or without diabetes, nor between male and female gender.

### Radiological findings

Clinical and biochemical characteristics of the cohort according to the presence or absence of VC is presented in Table 2. Vascular calcification was detected in 38 patients (67.9%) by lumbar aorta X-ray (Kauppila method) and in 36 patients (64.3%) by pelvic and hand X-rays (Adragão method). Median Kauppila and Adragão scores were 3 (IQR 0–6) and 1 (IQR 0–3), respectively. Inter-rater agreement was very good with a weighted Kappa of 0.83. The scores were moderately correlated with each other (*r* = 0.658, *P*<0.001). Patients with VC were older than their non-VC counterparts, but the difference was not statistically significant. Nonetheless, a positive correlation was noted between age and Kauppila score (*r* = 0.29, *P* = 0.04). The proportion of male subjects with VC on Kauppila score was significantly higher than that of females (86.8% vs 56.3%, *P* = 0.014). Patients presenting with VC were more likely to have diabetes than those without VC (76.3% vs 31.3%, *P* = 0.002, for Kauppila, and 74.3% vs 40.0%, *P* = 0.012, for Adragão index).

**Table 2:** Demographic, clinical and laboratory characteristics of the study population according to the presence or absence of vascular calcification.

		KS = 0 (*n* = 16, 28.6%)	KS≥1 (*n* = 38, 67.9%)	*P* value	AS = 0 (*n* = 20, 35.7%)	AS≥1 (*n* = 35, 62.5%)	*P* value
**Clinical**							
	Age (yr)	61.3 (13.3)	67.5 (6.8)	0.251	62.1 (12.7)	67.8 (7.4)	0.158
	Male (%)	56.3	86.8	0.014	70.0	82.9	0.267
	Diabetes (%)	31.3	76.3	0.002	40.0	74.3	0.012
	Body mass index (Kg/m^2^)	26.8 (3.9)	29.1 (4.2)	0.057	27.2 (3.9)	28.5 (4.2)	0.241
	SBP (mmHg)	132 (11)	136 (11)	0.111	134 (13)	136 (11)	0.706
	DBP (mmHg)	80 (7)	79 (9)	0.924	80 (8)	79 (9)	0.902
**Biochemistry**							
	Creatinine (mg/dL)	2.38 (0.41)	2.25 (0.42)	0.421	2.38 (0.39)	2.26 (0.43)	0.552
	GFR (mL/min/1.73 m^2^)	26.6 (5.9)	29.0 (6.9)	0.343	27.4 (5.9)	28.5 (6.9)	0.617
	Haemoglobin (g/dL)	12.8 (1.6)	13.2 (2.0)	0.532	13.4 (1.9)	12.8 (1.7)	0.558
	Albumin (g/dL)	4.20 (0.31)	4.14 (0.36)	0.884	4.24 (0.30)	4.11 (0.37)	0.765
	Bicarbonate (mmol/L)	24.1 (2.8)	25.5 (3.2)	0.257	24.0 (2.6)	25.9 (3.5)	0.152
	C-reactive protein (mg/L)	1.8 (1.0, 3.6)	3.6 (1.6, 6.5)	0.086	2.0 (1.2, 4.8)	3.5 (1.3, 7.3)	0.361
	LDL cholesterol (mg/dL)	95 (37)	93 (27)	0.849	89 (37)	96 (26)	0.203
	BNP (pg/mL)	41 (18, 52)	41 (20, 104)	0.440	21 (19, 53)	43 (30, 109)	0.147
	Calcium (mg/dL)	9.3 (0.4)	9.4 (0.5)	0.167	9.3 (0.4)	9.4 (0.5)	0.217
	Phosphorus (mg/dL)	3.7 (0.6)	3.4 (0.5)	0.162	3.6 (0.7)	3.5 (0.6)	0.586
	Magnesium (mEq/L)	1.72 (0.26)	1.60 (0.21)	0.220	1.68 (0.26)	1.64 (0.24)	0.942
	25(OH)D (ng/mL)	19 (13, 24)	14 (9, 22)	0.334	20 (12, 22)	14 (9, 22)	0.309
	ALP (U/L)	68 (57, 102)	81 (63, 103)	0.867	74 (57, 105)	84 (64, 103)	0.663
	iPTH (pg/mL)	119.4 (48.4, 165.9)	86.3 (49.9, 133.0)	0.602	97.4 (52.3, 197.1)	96.4 (49.0, 135.4)	0.868
	FGF23 (pg/mL)	24.4 (16.9, 58.3)	25.5 (16.3, 37.4)	0.693	33.1 (19.8, 45.1)	25.0 (15.7, 36.9)	0.271
	Sclerostin (pmol/L)	44.0 (32.6, 59.7)	61.9 (40.3, 85.8)	0.039	40.7 (29.2, 59.1)	63.9 (44.9, 97.9)	0.005
	DKK1 (pg/mL)	746 (586, 988)	794 (601, 1063)	0.550	716 (604, 817)	871 (599, 1146)	0.113
	sRANKL (pmol/L)	2.76 (2.41, 5.82)	2.76 (1.76, 4.23)	0.543	2.82 (1.82, 3.41)	2.71 (1.70, 5.46)	0.692
	Osteoprotegerin (pg/mL)	1327 (1025, 1749)	1422 (1169, 1638)	0.654	1146 (1023, 1426)	1464 (1308, 1750)	0.007
	Urine calcium (mg/day)^a^	68 (36, 108)	72 (38, 98)	0.960	76 (36, 108)	68 (36, 99)	0.437
	Urine phosphate (mg/day)^a^	667 (503, 719)	740 (559, 826)	0.374	667 (496, 776)	736 (566, 841)	0.389
	Urine protein (mg/day)^a^	770 (320, 2400)	565 (190, 2192)	0.508	1470 (335, 2550)	565 (200, 1808)	0.270

SBP, systolic blood pressure; DBP, diastolic blood pressure; GFR, glomerular filtration rate estimated by CKD-EPI equation; iPTH, intact parathyroid hormone; ALP, alkaline phosphatase; 25(OH)D, 25 hydroxyvitamin D; LDL, low-density lipoprotein; BNP, brain natriuretic peptide; sRANKL, soluble receptor activator of nuclear factor-κB ligand; DKK1, Dickkopf-1; FGF23, fibroblast growth factor-23; KS, Kauppila score; AS, Adragão score.

Data are reported as mean (SD) for normally distributed variables, median (interquartile range) for non-normally distributed variables, or percentage for categorical variables. P values were calculated using Mann-Whitney test for continuous variables and Chi-square test for categorical variables.

^a^Twenty-four-hour urine collections were adjusted for adequacy using urinary creatinine excretion.

Vascular calcification status did not differ significantly between CKD stages. Likewise, no correlation was found between GFR and both VC scores. Patients with VC had significantly higher plasma sclerostin levels than those without VC. Plasma OPG levels were also significantly higher in patients with VC, but only on Adragão score. No association was found with the remaining bone biomarkers.

**Fig 1:**
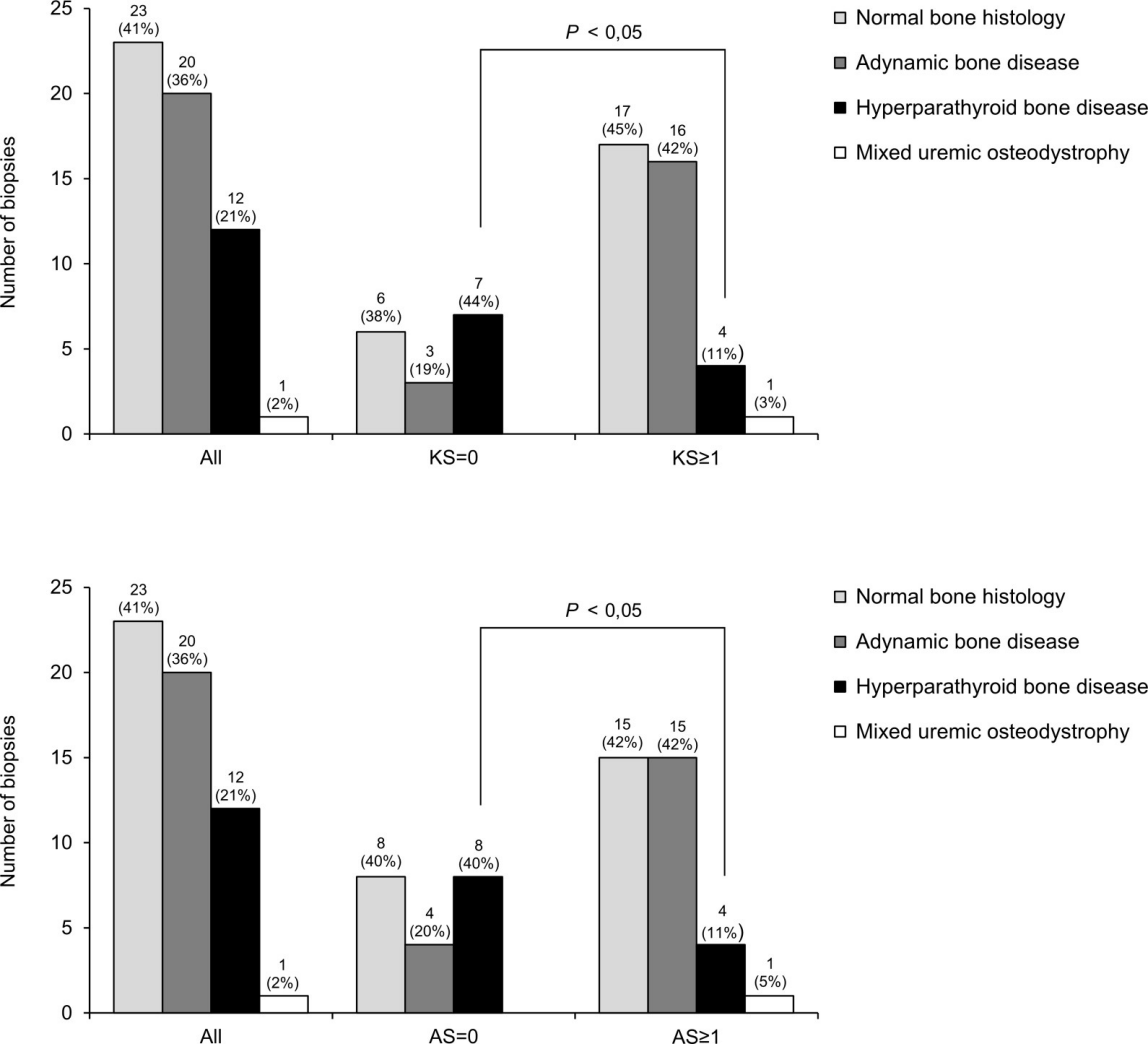
Distribution of bone histological patterns in relation to vascular calcification status.

The relative prevalence of bone histological forms differed in accordance with the absence or presence of VC (Fig 1). Non-VC patients were significantly more likely to present with hyperparathyroid bone disease than those with VC both on Kauppila (43.8% vs 11.1%, *P* = 0.006) and Adragão score (40.0% vs 11.4%, *P* = 0.016). Conversely, the prevalence of adynamic bone disease was higher in patients with VC than those without VC, but the difference was not statistically significant between groups.

Histomorphometric measurements as a function of VC status are shown in Table 3. Patients with VC had a significantly lower BFR/BS than those without VC. Bone volume/total volume was also lower in the former group, but the difference was not statistically significant. The remaining static and dynamic parameters did not differ between groups as well.

**Table 3:** Bone histomorphometric parameters according to vascular calcification status.

	All (*n* = 56)	KS = 0 (*n* = 16, 28.6%)	KS≥1 (*n* = 38, 67.9%)	*P* value	AS = 0 (*n* = 19, 33.9%)	AS≥1 (*n* = 36, 64.3%)	*P* value
BFR/BS (μm^3^/μm^2^/yr)	15.56 (8.56, 38.18)	38.24 (13.98, 41.28)	9.99 (5.39, 21.14)	0.019	38.24 (15.57, 40.69)	9.98 (4.69, 19.37)	0.004
Ob.S/BS (%)	0.41 (0.11, 0.91)	0.73 (0.11, 2.14)	0.34 (0.10, 0.75)	0.133	0.59 (0.14, 2.08)	0.34 (0.08, 0.75)	0.143
Oc.S/BS (%)	0.24 (0, 0.53)	0.25 (0, 0.72)	0.15 (0, 0.40)	0.664	0.31 (0.07, 0.69)	0.15 (0, 0.39)	0.177
OS/BS (%)	13.21 (6.40, 21.31)	17.51 (13.07, 24.87)	9.89 (5.94, 18.14)	0.096	17.01 (10.68, 26.17)	11.09 (6.02, 18.98)	0.074
ES/BS (%)	2.75 (1.51, 4.11)	2.53 (1.91, 5.77)	2.77 (1.36, 4.01)	0.776	3.42 (2.24, 7.71)	2.39 (1.16, 3.69)	0.069
Mlt (d)	22.55 (12.47)	18.39 (8.57)	25.07 (13.76)	0.281	18.99 (12.16)	24.76 (12.44)	0.193
OV/BV (%)	2.57 (1.15, 3.30)	2.88 (1.73, 3.95)	2.30 (1.05, 3.21)	0.384	2.85 (1.33, 3.84)	2.44 (1.06, 3.27)	0.612
O.Th (μm)	6.44 (1.72)	6.38 (1.59)	6.47 (1.86)	0.472	6.60 (1.62)	6.35 (1.83)	0.431
BV/TV (%)	19.09 (6.81)	21.34 (3.44)	17.74 (7.84)	0.532	22.44 (4.13)	16.98 (7.34)	0.074

BFR/BS, bone formation rate/bone surface; Ob.S/BS, osteoblast surface/bone surface; Oc.S/BS, osteoclast surface/bone surface; OS/BS, osteoid surface/bone surface; ES/BS, eroded surface/bone surface; Mlt, mineralization lag time; OV/BV, osteoid volume/bone volume; O.Th, osteoid thickness; BV/TV, bone volume/total volume.

Data are reported as mean (SD) for normally distributed variables and median (interquartile range) for non-normally distributed variables. P values were calculated using Mann-Whitney test.

Binary logistic regression analysis was performed in order to assess the impact on the likelihood of VC of the variables found to be significant in the univariate analysis (Table 4). The model showed that BFR/BS, adjusted for sex, diabetes, plasma sclerostin and plasma OPG, was independently associated with the presence of VC, assessed by either Kauppila or Adragão score (*P* = 0.035, odds ratio (OR) = 0.90, 95% confidence interval (CI) 0.82–0.99 and *P* = 0.017, OR = 0.88, 95% CI 0.79–0.98; respectively).

**Table 4:** Multivariable logistical regression analysis for prediction of vascular calcification.

	KS≥1				AS≥1			
	β	OR	95% CI	*P* value	β	OR	95% CI	*P* value
Sex (male versus female)	2.747	15.59	0.971–250.1	0.052	1.337	3.806	0.301–48.29	0.303
Diabetes (yes versus no)	1.792	6.001	0.499–72.11	0.158	2.409	11.12	0.948–130.5	0.055
Sclerostin (pmol/L)	0.021	1.021	0.983–1.062	0.283	0.011	1.011	0.976–1.047	0.541
Osteoprotegerin (pg/mL)	0.001	1.001	0.998–1.004	0.503	0.002	1.002	0.999–1.006	0.116
BFR/BS (μm^3^/μm^2^/yr)	- 1.020	0.903	0.821–0.993	0.035	- 0.128	0.879	0.792–0.977	0.017

KS, Kauppila score; AS, Adragão score; OR, odds ratio; CI, confidence interval; BFR/BS, bone formation rate/bone surface.

## Discussion

Vascular calcification is a well-described complication of chronic kidney disease and strongly associates with increased risk for cardiovascular events and death [1, 28]. Though not yet fully understood, VC is a complex cell-mediated process that shares many similarities to bone formation and mineralization [4].

Semi-quantitative scoring systems obtained through plain X-rays of abdominal aorta (Kauppila score) or pelvis and hands (Adragão score) show good correlation with computed tomography (CT), which is the gold standard tool for estimation of VC [12, 23]. They are inexpensive, widely available, easy to interpret and result in low radiation exposure. Consequently, Kidney Disease Improving Global Outcomes (KDIGO) guidelines consider that plain radiographs are a reasonable alternative to CT-based imaging for VC evaluation [8]. Kauppila and Adragão scores are associated with higher cardiovascular mortality in dialysis patients and, more recently, the latter was reported to predict death in patients not yet on dialysis [24–26].

Vascular calcification was detected in roughly two-thirds of our cohort, a result comparable to previous reports [26]. Such a high prevalence suggests that VC starts early in the course of CKD, supporting the KDIGO recommendation for screening these patients [8]. The VC group had a higher proportion of males and presented with a higher prevalence of diabetes than the non-VC group. A positive correlation was also observed between age and Kauppila score. These findings were not surprising, since age, sex and diabetes have long been identified has traditional risk factors for VC [29, 30].

Sclerostin, an osteocyte-derived glycoprotein, is an antagonist of the canonical Wnt signaling pathway and has the physiological role to reduce bone formation [31]. Sclerostin circulating levels are increased in CKD patients [32] and in most studies are positively associated with VC [33]. However, it is not yet clear whether raised sclerostin concentrations in CKD patients result from increased production, reduced elimination, or both [34]. In our study, the univariate analysis showed that VC subjects had significantly higher plasma sclerostin levels than non-VC counterparts. Claes *et al* [35] reported the same positive association in a cohort of predialysis patients, in which aortic calcification was ascertained through Kauppila method. However, in the multivariable analysis, lower, not higher, sclerostin levels were identified as independent determinants of VC. Since sclerostin is expressed not only by bone but also calcifying vascular smooth muscle cells, the authors hypothesized that vessels might have an important contribution to sclerostin circulating levels, performing a counterregulatory role to inhibit the progression of VC. Additional studies are needed in order to clarify the relationship between sclerostin and vascular disease in CKD patients.

Osteoprotegerin is another biomarker that has become important for its potential role in vascular disease and calcification [36]. Osteoprotegerin is a glycoprotein expressed by osteoblasts that binds and inhibits RANKL, thus suppressing osteoclastogenesis and bone resorption [37]. Paradoxically, although thought to have a protective role against VC, OPG levels increase with CKD progression [38]. As with sclerostin, this may represent a compensatory mechanism to inhibit cardiovascular calcification [39]. Nevertheless, it remains to be established to what extent renal impairment contributes to increased OPG concentrations [38]. In our cohort, patients with a positive Adragão score had significantly higher plasma OPG levels in the univariate analysis than those without VC. A positive correlation between circulating OPG and CAC, assessed by CT, has been described in both dialysis and predialysis patients [40, 41]. However, to the best of our knowledge this is the first report of an association between OPG and a simple VC score in predialysis patients. A positive correlation between RANKL/OPG ratio and VC has also been described in non-CKD patients [42], but we found no association with this potential biomarker.

In our study, the multivariable analysis showed that BFR/BS was independently associated with the presence of VC. The relationship between renal osteodystrophy and VC in predialysis CKD has been reported by Tomiyama *et al* [13]. In a group of 50 patients not yet on dialysis, the authors found a high prevalence of CAC and showed that low BFR/BS, adjusted for age, sex and diabetes, was independently associated with the presence of coronary calcification. There are some studies that have been conducted in dialysis patients. London *et al* [9] reported that high arterial calcification scores on ultrasound evaluation associated with bone histomorphometric values suggestive of adynamic bone disease, in a cohort of patients undergoing hemodialysis. This association was confirmed prospectively in a study by Barreto *et al* [10], which showed a negative correlation between CAC and trabecular bone volume in a group of hemodialysis patients, and that low-turnover bone status was the only independent predictor for CAC progression. Adragão *et al* [11, 12] have also reported an association between low bone volume and increased VC by both computed tomography and plain X-ray in patients on hemodialysis. Research on the relationship between renal osteodystrophy and VC could be promoted through the use of simple radiographs, since they are inexpensive and readily available, as compared with CT-based techniques.

Traditional risk factors do not completely account for the development of VC in CKD patients [1]. Our results add to the evidence that renal osteodystrophy is emerging as an important contributing factor for the high cardiovascular risk associated with CKD. Therefore, intervention might be needed in order to prevent or treat low bone turnover disease in early CKD, with the aim of avoiding the occurrence and progression of cardiovascular calcifications [7].

Our study has some limitations. The cross-sectional nature of the study does not allow to infer a relationship of causality from the reported associations. Additionally, the number of patients included was relatively modest, which may have impaired the ability to detect further significant associations in the regression analysis. However, to the best of our knowledge, this is the first report of an association between bone histomorphometric parameters and simple VC scores in predialysis patients. Moreover, no participant had previously been on drugs known to interfere with bone metabolism, thus ruling out a potentially relevant confounding factor.

In conclusion, we found that low BFR/BS was independently associated with the occurrence of VC in a cohort of patients not yet on dialysis, suggesting that low bone activity may be a non-traditional risk factor for cardiovascular disease in CKD patients. Prospective studies in the predialysis population are required in order to evaluate the impact of preventing and treating renal osteodystrophy on the development and progression of VC.

## Supporting information

S1 Table Demographic, clinical and laboratory characteristics of the study population according to diabetes status. (DOCX)

S1 Dataset (SAV)
