# Supplementary material for: Low bone turnover is associated with plain X-ray vascular calcification in predialysis patients
Source: PLoS One. 2021 Oct 13;16(10):e0258284. doi: 10.1371/journal.pone.0258284 (PMC8513829; doi:10.1371/journal.pone.0258284)
Supplement: S1 Table — (DOCX) [file pone.0258284.s001.docx]

**S1 Table. Demographic, clinical and laboratory characteristics of the study population according to diabetes status.**

|  |  | **All**  **(*n*=56)** | **With diabetes**  **(*n*=35, 62.5%)** | **Without diabetes**  **(*n*=21, 37.5%)** | ***P* value** |
| --- | --- | --- | --- | --- | --- |
| **Clinical** | |  |  |  |  |
|  | Age (yr) | 65.7 (9.8) | 66.9 (8.1) | 63.7 (12.0) | 0.503 |
|  | Male (%) | 78.6 | 80.0 | 76.2 | 0.748 |
|  | Body mass index (Kg/m^2^) | 28.4 (4.3) | 28.4 (4.2) | 27.6 (3.9) | 0.282 |
|  | SBP (mmHg) | 136 (11) | 137 (11) | 134 (11) | 0.471 |
|  | DBP (mmHg) | 78 (8) | 80 (9) | 80 (8) | 0.986 |
| **Biochemistry** | |  |  |  |  |
|  | Creatinine (mg/dL) | 2.32 (0.43) | 2.31 (0.46) | 2.31 (0.38) | 0.370 |
|  | GFR (mL/min/1.73 m^2^) | 27.8 (6.8) | 28.1 (7.1) | 27.4 (6.4) | 0.536 |
|  | Haemoglobin (g/dL) | 13.0 (1.7) | 12.8 (1.9) | 13.4 (1.4) | 0.282 |
|  | Albumin (g/dL) | 4.16 (0.35) | 4.21 (0.38) | 4.06 (0.21) | 0.018 |
|  | Bicarbonate (mmol/L) | 25.2 (3.3) | 25.7 (3.2) | 24.2 (3.2) | 0.573 |
|  | C-reactive protein (mg/L) | 2.0 (0.8, 6.1) | 3.6 (1.0, 8.0) | 1.2 (0.5, 1.6) | 0.011 |
|  | LDL cholesterol (mg/dL) | 94 (30) | 92 (25) | 97 (39) | 0.936 |
|  | BNP (pg/mL) | 43 (21, 106) | 63 (29, 139) | 31 (19, 49) | 0.021 |
|  | Calcium (mg/dL) | 9.3 (0.5) | 9.2 (0.5) | 9.4 (0.3) | 0.602 |
|  | Phosphorus (mg/dL) | 3.5 (0.6) | 3.7 (0.5) | 3.3 (0.6) | 0.037 |
|  | Magnesium (mEq/L) | 1.65 (0.24) | 1.67 (0.27) | 1.64 (0.18) | 0.637 |
|  | 25(OH)D (ng/mL) | 16 (9, 22) | 13 (9, 21) | 21 (14, 25) | 0.058 |
|  | ALP (U/L) | 81 (63, 102) | 79 (64, 107) | 81 (57, 94) | 0.606 |
|  | iPTH (pg/mL) | 93.3 (50.6, 151.4) | 98.5 (67.4, 204.2) | 80.4 (41.6, 145.6) | 0.257 |
|  | FGF23 (pg/mL) | 25.5 (16.3, 38.4) | 26.7 (16.3, 40.7) | 23.3 (11.3, 36.3) | 0.344 |
|  | Sclerostin (pmol/L) | 57.6 (38.6, 72.8) | 61.1 (43.4, 86.2) | 39.3 (31.4, 58.9) | 0.035 |
|  | DKK1 (pg/mL) | 800 (276) | 1150 (1081) | 929 (675) | 0.350 |
|  | sRANKL (pg/mL) | 2.66 (1.69, 3.58) | 2.80 (1.74, 5.26) | 2.48 (1.62, 3.06) | 0.330 |
|  | Osteoprotegerin (pg/mL) | 1385 (1120, 1717) | 1429 (1152, 1698) | 1270 (1057, 1721) | 0.350 |
|  | Urine calcium (mg/day)^a^ | 70 (36, 99) | 78 (39, 113) | 60 (35, 80) | 0.169 |
|  | Urine phosphate (mg/day)^a^ | 693 (556, 797) | 742 (563, 894) | 636 (478, 728) | 0.087 |
|  | Urinary protein (mg/day)^a^ | 720 (255, 2287) | 780 (235, 2690) | 30 (250, 2110) | 0.461 |

SBP, systolic blood pressure; DBP, diastolic blood pressure; GFR, glomerular filtration rate estimated by CKD-EPI equation; LDL, low-density lipoprotein; BNP, brain natriuretic peptide; 25(OH)D, 25 hydroxyvitamin D; ALP, alkaline phosphatase; iPTH, intact parathyroid hormone; FGF23, fibroblast growth factor-23; DKK1, Dickkopf-1; sRANKL, soluble receptor activator of nuclear factor-κB ligand.

Data are reported as mean (SD) for normally distributed variables, median (interquartile range) for non-normally distributed variables, or percentage for categorical variables. *P* values were calculated using Mann-Whitney test for continuous variables and Chi-square test for categorical variables.

^a^Twenty-four hour urine collections were adjusted for adequacy using urinary creatinine excretion.
